# Selective vulnerability of two dopamine neuron populations with distinct spatial distribution in the human substantia nigra

**DOI:** 10.1002/alz70855_098851

**Published:** 2025-12-23

**Authors:** Shinnosuke Yamada, Huifangjie L. Farsad, Wei Feng, Ibrahim Olabayode Saliu, Erin E. Franklin, Richard J. Perrin, Nigel J Cairns, John C. Morris, Joel S. Perlmutter, Jinbin Xu, Guoyan Zhao

**Affiliations:** ^1^ Washington University School of Medicine, St. Louis, MO, USA; ^2^ Washington University, St. Louis, MO, USA; ^3^ Department of Neurology, Washington University in St. Louis School of Medicine, St. Louis, MO, USA; ^4^ Univeristy of Exeter, Exeter, MO, United Kingdom; ^5^ Knight Alzheimer Disease Research Center, St. Louis, MO, USA; ^6^ Department of Neurology, Washington University School of Medicine, St. Louis, MO, USA; ^7^ Department of Radiology, Washington University School of Medicine, St. Louis, MO, USA; ^8^ Washington University School of Medicine, St Louis, MO, USA

## Abstract

**Background:**

Neuropathology of Lewy bodies is reported in Parkinson disease (PD), Parkinson disease dementia (PDD) and dementia with Lewy bodies (DLBD), and the latter two disorders comprise Lewy body dementias (LBDs). LBDs and Alzheimer's disease (AD) are the most common neurodegenerative dementias with the commonality of protein deposition but distinct pathological and clinical characteristics.

**Method:**

We performed snRNA‐seq on the substantia nigra (SN) tissues obtained from 45 subjects with AD, PD, PDD, or DLBD, and cognitively normal controls. We compared our data with three published snRNA‐seq data of SN derived from subjects with PD or PDD and cognitively normal controls by Wang et al., 2022, Kamath et al. 2022, and Martirosyan et al. 2024 through uniform data analysis using the same analysis approaches and parameters. We performed MERFISH spatial transcriptomics and immunohistochemical staining to investigate spatial distribution of neuronal populations and their selective vulnerability.

**Result:**

We identified 9 neuronal subpopulations in our data including two Dopamine (DA) neuron populations: SOX6+ vs. SOX6‐ DA neurons. SOX6+ DA neurons, RORB+, and FAT2+ neurons were shared by all four datasets whereas the SOX6‐ DA neurons were shared only by the Martirosyan et al. data. Additionally, other neuronal populations were shared by some but not all datasets and each dataset had their own unique neuronal population suggesting the regional differences among the four datasets. Differentially expressed gene (DEG) analysis in each disease condition revealed that DEGs of DA neurons in AD overlapped significantly with those of PD and LBDs. However, *ALDH1A1* expression was oppositely regulated in AD versus in PD and LBDs, which may contribute to selective vulnerability of DA neurons in PD and LBDs. We demonstrated that SOX6‐ DA neurons were more vulnerable than the SOX6+ DA neurons and they had distinct spatial distributions in human SN with SOX6‐ DA neurons located at ventral SN. Pathway analysis revealed vast differences in gene dysregulation in the corresponding neuronal populations among the four datasets.

**Conclusion:**

We report spatial heterogeneity of both cell composition and gene dysregulation in PD and LBDs, unveiling molecular mechanisms underlying selective neuronal and regional vulnerability in the human SN.

